# Advancing Glaucoma Care in Bangladesh With Minimally Invasive Glaucoma Surgery

**DOI:** 10.7759/cureus.60905

**Published:** 2024-05-23

**Authors:** Md Iftekher Iqbal, Fariah Osman

**Affiliations:** 1 Ophthalmology, Bangladesh Eye Hospital, Dhaka, BGD; 2 Glaucoma, Ispahani Islamia Eye Institute and Hospital, Dhaka, BGD; 3 Ophthalmology, Ispahani Islamia Eye Institute and Hospital, Dhaka, BGD

**Keywords:** bangladesh, primary open-angle glaucoma (poag), trabex+, migs, minimally invasive glaucoma surgery

## Abstract

Minimally invasive glaucoma surgery (MIGS) is a cutting-edge approach to treating glaucoma that provides a range of techniques and technology to reduce intraocular pressure (IOP). An 80-year-old man with visually significant cataracts and primary open-angle glaucoma (POAG) underwent combined cataract surgery and TrabEx+ (MicroSurgical Technology, Washington, United States) in his left eye, a unique type of MIGS, as we described in this study. Over the one-year follow-up, this patient showed improved visual function with well-controlled IOP without anti-glaucoma medications.

## Introduction

Primary open-angle glaucoma (POAG) and many other types of secondary glaucoma are both marked by a rise in the resistance to the outflow of aqueous humor and a difference in pressure, especially across the trabecular meshwork (TM) [1]. Thus, surgical methods to remove or bypass the TM can lower intraocular pressure (IOP) and lessen the chance of developing progressive glaucomatous optic neuropathy. Under the category of minimally invasive glaucoma surgery (MIGS), there are various ab-interno trabeculectomy procedures [2].

MIGS presents fewer risks and provides an opportunity to reduce IOP and medication requirements in comparison to traditional filtering surgery [3]. It generally refers to glaucoma operations that have the following qualities: little anatomical disruption with biocompatible materials, ab-interno with no damage to conjunctival tissue, good efficacy, a quick recovery period, and a favorable safety profile [3,4].

Ab-interno trabeculotomy operations that remove TM and protect the anterior chamber (AC) employ different approaches. TrabEx+ (MicroSurgical Technology, Washington, United States) is a one-time-use, serrated trapezoidal dual-bladed instrument designed for irrigating goniectomy through a corneal microincision. Its goal is to remove three to six clock hours of TM. The handpiece features irrigation and aspiration (IA) ports that can be attached to ordinary phacoemulsification machines. This makes it possible to stabilize the AC without using ocular viscoelastic devices (OVDs) [2,5].

We described in this study a case of bilateral POAG with visually significant cataract that was treated with phacoemulsification with TrabEx+ in his left eye (LE), and a one-year follow-up period ensued. As far as our understanding goes, there is no publication from Bangladesh that specifically addresses the MIGS or TrabEx+.

## Case presentation

An 80-year-old Bangladeshi gentleman was referred by his primary ophthalmologist to a glaucoma specialist for additional evaluation and management of this patient as a glaucoma suspect. He was visited with blurry vision for more than a year, which got worse over the last few months.

According to the patient, he has been diabetic for seven years and is on oral hypoglycemic medication with good control of diabetes and no positive family history of glaucoma or other risk factors. He also mentioned the uneventful appendectomy surgery a few years ago.

On ophthalmological evaluation of the patient, his best-corrected visual acuity (BCVA) was 6/24 in the right eye (RE) and 6/36 in the LE, measured with the Snellen chart. IOP with a Goldmann applanation tonometer showed marked elevation in both eyes: 26 mmHg in the RE and 22 mmHg in the LE.

Slit-lamp biomicroscopy (SLBM) of both eyes revealed a deep AC, nuclear cataract (grade 4) with few cortical changes in the lenses, and Shaffer’s grade-4 AC angle in gonioscopy visible with Volk 4-mirror goniolens (Volk Optical, Inc., Ohio, United States).

**Figure 1:**
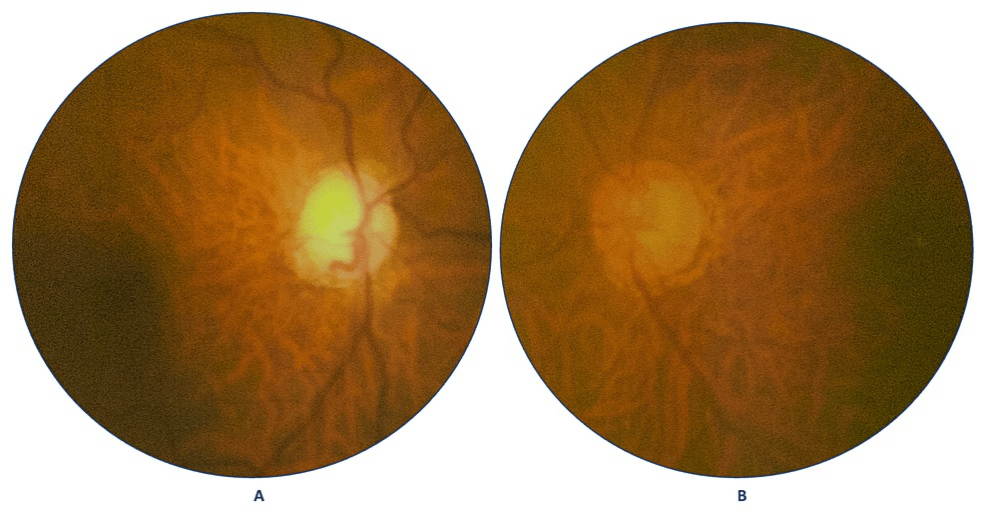
Color fundus images of the right eye (A) and the left eye (B), respectively.

During dilated fundus examination with a Volk 90D (Volk Optical, Inc., Ohio, United States) condensing lens, the optic nerve head (ONH) evaluation of the RE (Figure 1A) showed an increased cup-to-disc ratio of 0.9:1, thinned neuroretinal rim (NRR), nasal shifting of the blood vessels, and decreased superficial papillary vessels with peripapillary atrophy (PPA), whereas the LE (Figure 1B) showed an increased cup-to-disc ratio of 0.6:1, inferior and superior thinning of the NRR, with PPA. Overall, the retinas of both eyes looked tigroid-like in appearance, with apparently normal-looking peripheral retinas and no notable features of diabetic retinopathy.

Clinically, this patient was diagnosed with a case of POAG with nuclear sclerosis (grade 4) in both eyes and prescribed combination eye drops of timolol maleate 0.5% and brimonidine tartrate 0.2% twice daily, along with brinzolamide 1% thrice daily in both eyes.

**Figure 2:**
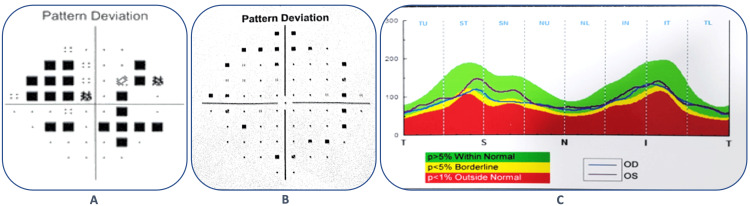
Pattern deviation from the Humphrey visual field analysis reports of the right eye (A) and the left eye (B); OCT-RNFL of both eyes (C). OCT: optical coherence tomography; RNFL: retinal nerve fiber layer

His Humphrey visual field analysis (Carl Zeiss, San Diego, USA) showed advanced glaucomatous field changes with a visual field index (VFI) of 76%, a mean deviation (MD) of -11.6 dB, and a pattern standard deviation (PSD) of 15.36 dB in the RE (Figure 2A), and early glaucomatous field changes with a VFI of 88%, a MD of -15.8 dB, and a PSD of 3.6 dB in the LE (Figure 2B). Optical coherence tomography (OCT) also showed significant retinal nerve fiber layer (RNFL) damages in both eyes, with an average RNFL thickness of 86 μm (RE) and 94 μm (LE), a superior RNFL thickness of 84 μm (RE) and 97 μm (LE), and an inferior RNFL thickness of 87 μm (RE) and 91 μm (LE) (Figure 2C).

**Figure 3:**
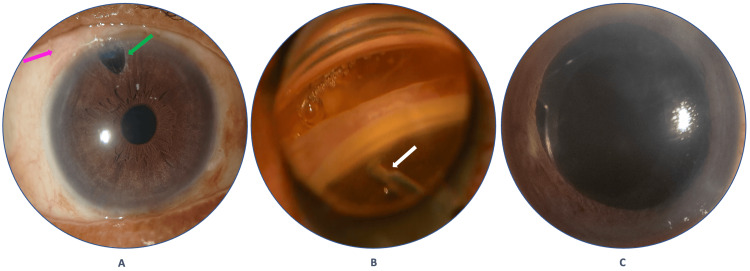
(A) Pseudophakic right eye with surgical peripheral iridectomy (green arrow) and a portion of the functioning bleb (pink arrow); (B) Left eye: intraoperative gonio view of the TrabEx+ (white arrow); (C) Left eye: 30-minute postoperative status showing no hyphema and well-centered IOL. IOL: intraocular lens

After proper counseling about the disease process and all the available treatment options for the patient, he underwent combined mitomycin-C (0.2 mg/mL) augmented trabeculectomy and cataract surgery with single-piece intraocular lens (IOL) implantation in the RE, which is now maintaining BCVA of 6/9 (-0.50 DS) with N6, and IOP of 14 mmHg without any antiglaucoma medications (AGM) for the last 13 months post-operatively. Figure 3A shows pseudophakic RE with a surgical peripheral iridectomy and part of the diffusely functioning bleb.

After one month of RE surgery, he underwent cataract extraction with TM-based MIGS with TrabEx+ in the LE, which was used for the first time in Bangladesh. Initially, standard cataract surgery (phacoemulsification) was done temporally with the implantation of a single-piece IOL in the capsular bag, then the surgical microscope was tilted 45° and the patient’s head was rotated away from the surgeon’s position for better visualization of the AC angle with the Volk surgical goniolens. Then, after proper alignment of the surgical goniolens over the cornea, aided by a viscoelastic substance, and proper identification of the TM, TrabEx+ was introduced into the AC, and TM-stripes were removed with the help of the serrated blade of the device for around five-clock hours of the AC angle (Figure 3B) and opened up the inner wall of the Schlemm’s canal. After aspiration of the refluxed blood and viscoelastic substance, the AC was formed, and the wound was hydrated with a balanced salt solution (BSS). Intracameral moxifloxacin 100 mcg in 0.1 mL was administered at the end of the surgery. After 30 minutes post-operatively, eye patching was removed, and the SLBM examination was performed to look for any hyphema or other immediate postoperative complications (Figure 3C). Then the patient was prescribed topical moxifloxacin 0.5% four times daily for a month, along with prednisolone acetate 1% hourly for a day, and then tapered over a month.

**Figure 4:**
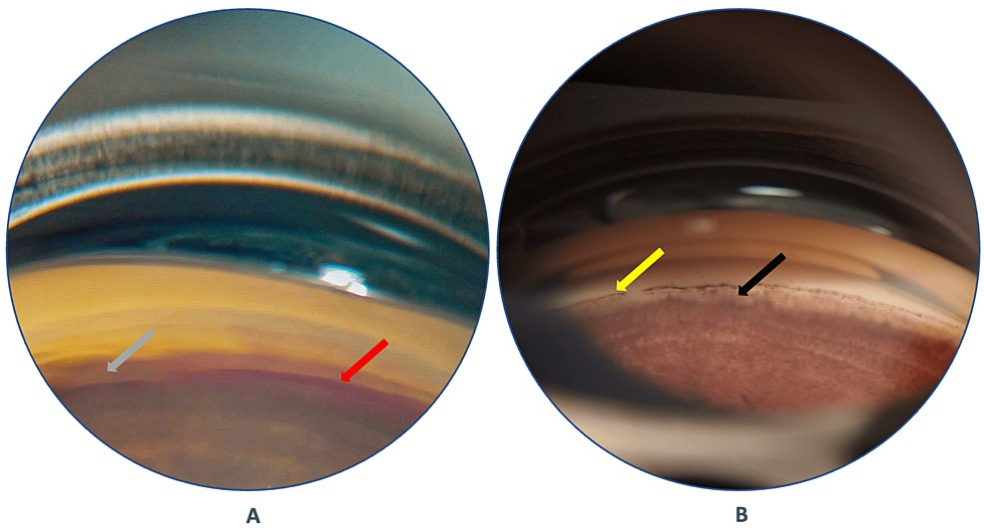
(A) Seventh POD gonio view of the left eye: blood in the Schlemm canal (red arrow) and wide ciliary body band (grey arrow); (B) Three-month POD gonio view of the left eye: wide open anterior chamber angle with Sampaolesi line (yellow arrow) and PAS (black arrow). POD: postoperative day

On his seventh postoperative day (POD) follow-up of the LE, his BCVA was 6/9, N6, with an IOP of 18 mmHg without any AGM. A gonioscopy revealed blood in the Schlemm’s canal with a wide open AC angle (Figure 4A). On the third postoperative month of the LE, he maintained the same visual function with an IOP of 14 mmHg, and gonioscopy showed a wide angle with the Sampaolesi line and a peripheral anterior synechiae (PAS) (Figure 4B).

**Figure 5:**
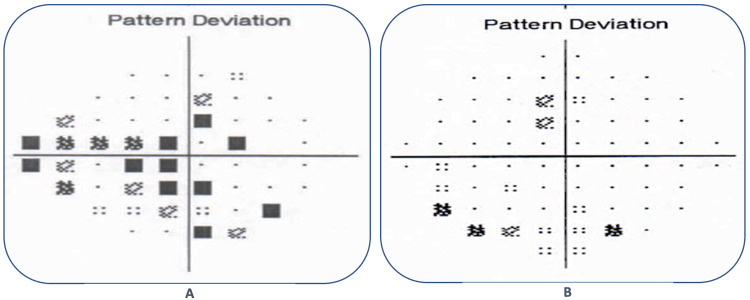
Pattern deviation from the Humphrey visual field analysis reports of the right eye (A) and the left eye, respectively, one year after surgery.

The patient was on regular follow-up, maintaining good vision and controlled IOP in both eyes over the last year, and at this point, his visual field showed improved visual functions (Figures 5A, 5B) with VFI 73%, MD -11.61 dB, and PSD 12.95 dB in the RE and VFI 92%, MD -7.77 dB, and PSD 1.60 dB in the LE.

## Discussion

Bangladesh is a developing country with a population of about 171.2 million [6]. According to the recent nationwide survey in Bangladesh by the Bangladesh Glaucoma Society (BGS), POAG was 78%, of which 83% was normal tension glaucoma in an age group of 35 years and older [7]. Previously, most glaucoma patients were presented in the advanced or late stage of the disease in our country, but due to the rapid development of eye care services, the availability of the latest diagnostic modalities, and mass awareness about glaucoma throughout the year, we can now detect and treat glaucoma in the early stage.

While MIGS’s ability to lower IOP is not as strong as that of traditional glaucoma procedures, it is nonetheless widely advised for managing mild to moderate glaucoma [8,9]. Yet, MIGS is not popular in Bangladesh, most probably due to advanced disease at presentation, the need for additional surgical goniolens with devices, which are costlier for the surgeons and will lead to a high operation charge for the patients, and the lack of availability of hands-on training. However, due to the earlier disease diagnosis, availability of sophisticated surgical microscopes, surgical gonio lenses and devices, and online resources, MIGS is getting attention from glaucoma specialists, especially the young glaucoma experts of the country these days. Here, we presented the first reported case of MIGS using TrabEx+, combined with phacoemulsification from Bangladesh.

Though they both employ cutting to remove TM, TrabEx+ and the Kahook Dual Blade (KDB) (New World Medical, Rancho Cucamonga, CA) differ in that TrabEx+ includes an integrated IA in the handpiece and a different blade design. There are additional similarities with the Trabectome (MicroSurgical Technology, Washington, USA), as both instruments use IA, but the Trabectome ablates TM with a bipolar current. The following three devices differ from previous methods that incise or burst TM rather than remove it: goniotomy, gonioscopy-assisted transluminal trabeculotomy (GATT), and Microhook (Inami & Co. Ltd., Tokyo, Japan) ab-interno trabeculotomy are some of these other techniques [2]. We used TrabEx+ in addition to cataract surgery in the LE of this case for moderate POAG with a visually significant cataract.

Although improvement in vision after glaucoma surgery is not anticipated, the goal of early surgery is to minimize the danger of further decline in visual function as well as the burden of medication. Many studies have shown that a significant IOP reduction in glaucoma patients can increase visual sensitivity since a 1985 study that reported improved eyesight in glaucoma patients receiving treatment [10]. However, there is some evidence suggesting that glaucoma-damaged retinal ganglion cells may experience a phase of reversible dysfunction prior to cell death [11]. Additionally, reversible alterations in the morphology of the ONH have been documented subsequent to a decrease in IOP [12]. Based on these observations, it is plausible that specific patients could potentially experience certain structural and functional enhancements. Over the one-year follow-up of our patient, his visual field analysis showed improved visual sensitivity, most probably due to the removal of the cataract, reduced IOP led to increased perfusion of the less-perfused nerve fibers of the ONH and RNFL, and previous orientation with the visual field testing method. However, there was no evidence of structural improvement in the OCT of ONH and RNFL in our patient.

## Conclusions

MIGS can be a risk-free, quick-recovery method for controlling IOP, reducing the need for medication, and improving the quality of life for glaucoma patients in countries like Bangladesh. However, to guarantee widespread adoption and optimal patient results, additional research, training, and accessibility activities are necessary.
